# Editorial: Neuroplasticity in multiple sclerosis

**DOI:** 10.3389/fneur.2025.1550152

**Published:** 2025-02-04

**Authors:** Yavor Yalachkov, Soheila Karimi-Abdolrezaee, Eleonora Tavazzi

**Affiliations:** ^1^Department of Neurology, University Hospital, Goethe-University, Frankfurt, Germany; ^2^Department of Physiology and Pathophysiology, Rady Faculty of Health Sciences, Spinal Cord Research Centre, University of Manitoba Winnipeg, Winnipeg, MB, Canada; ^3^Buffalo Neuroimaging Analysis Center, University at Buffalo, Buffalo, NY, United States

**Keywords:** multiple sclerosis, neuroplasticity, neuroprotection, neuroregeneration, remyelination, BDNF, disability improvement, TMS

In the past several years, multiple sclerosis (MS) research has achieved significant progress in identifying novel disease mechanisms, diagnostic markers and therapeutic targets. The concept of NEDA-3 (no evidence of disease activity: no relapses, no disability progression, no MRI activity) seems nowadays realistic for a significant proportion of the patients on immune therapies. “Hit hard and early” or “hit smart and early” are common concepts which are often discussed as early treatment strategies.

Although the efficacy of currently available disease modifying therapies is indisputable, their effectiveness on both CNS-intrinsic neuroinflammation, neurodegeneration and neural repair is far from satisfactory. While MS symptoms can be treated, there are minimal treatment strategies to revert neurological disability or to effectively prevent gradual, relapse-independent disability accumulation (PIRA, progression independent of relapse activity). In this context, one of the main unmet needs is a better understanding of both the mechanisms driving progressive tissue loss, and the neuroplastic processes that attempt to compensate for the functional and structural tissue damage. Several studies have explored the pathways that regulate recovery from relapses and the processes that might be responsible for reverting disability progression. Investigating pharmacological and non-pharmacological interventions that facilitate remyelination and brain reorganization has resulted in several promising approaches.

While neuroplasticity can be described as a general term addressing the ability of the nervous system to adapt and modify both its structure and function in response to stimuli and experience, in this topic focused on the neuroplasticity of the central nervous system (CNS) in patients with MS and animal models of MS. We were particularly interested which molecular mechanisms determine clinical improvement after CNS demyelination; how the potential of the respective neuroplasticity processes can be measured—in both clinical and imaging/laboratory terms; which molecules or processes might serve as protective factors against demyelination or facilitate more efficient recovery; and last but not least whether immune therapies differ in their remyelination potential and how it can be maximized.

One less known but apparently already gaining attention non-pharmacological intervention in MS is photobiomodulation. Filho et al. systematically reviewed the evidence for the neuroprotective effects of this intervention. They were able to show that photobiomodulation affects CNS markers linked to inflammation, oxidative stress, and apoptosis. This method demonstrated also some improvements in motor, sensory, and cognitive functions in MS patients. Importantly, no study reported adverse effects. Thus, although the evidence seems currently limited, future exploration of photobiomodulation e.g. as a part of rehabilitation programmes seems warranted.

Another important contribution to the topic of rehabilitation and plasticity in MS was reported by Petracca et al. Here, MS patients were assigned to either a 6-week telerehabilitation or a 6-week onsite rehabilitation. The entire sample benefited from the treatments, with significant improvements observed at both group and individual levels across all measured domains (quality of life, fatigue, information processing speed, balance). Thus, telerehabilitation seems to be at least as powerful a tool as onsite rehabilitation and this might apply for most of the patients—in this study, the cohort covered a broad range of physical disability (EDSS ranging from 2 to 6.5).

Maiworm systematically reviewed the evidence for the role of brain-derived neurotrophic factor (BDNF) in neuroprotection and neuroplasticity. The author concluded that the current evidence remains inconclusive. There seems to be some beneficial effect of BDNF in MS, as studies reporting positive effects outweighed studies assuming detrimental effects of BDNF. Furthermore, studies regarding the Val66Met polymorphism have not conclusively determined whether this is a protective or harmful factor in MS. Most studies hypothesized a protective effect through modulation of BDNF secretion and anti-inflammatory effects with different effects in healthy controls and patients with MS, possibly due to the pro-inflammatory milieu in MS.

Zamali et al. investigated the role of IL-22 in myelination by employing a Cuprizone mouse model of experimental autoimmune encephalomyelitis. In Cuprizone-treated mice, application of IL-22 significantly improved motor and behavioral performance and robustly promoted remyelination in the corpus callosum. Additionally, IL-22 administration led to a significant elevation in myelin basic protein (MBP) transcription. Thus, the authors suggested a role for IL-22 in the pathophysiology of MS, particularly in supporting the process of remyelination.

Balloff et al. employed transcranial magnetic stimulation (TMS) and in particular the application of quadripulse stimulation to study the predictive value of synaptic plasticity for functional decline in MS. This method is known for its ability to induce both long-term potentiation and depression (LTP and LTD) in healthy subjects. The investigated patient cohort showed no clinically relevant change in any functional outcome over the observation period. However, MS patients who experienced clinically relevant decline in manual dexterity and/or visuospatial learning and memory had significantly lower levels of synaptic plasticity at baseline compared to those without such decline. Similar results were achieved also for visuospatial learning and memory. Thus, individual variability in plasticity might be relevant for the functional outcome over time.

Martin and Schneider focused in a mini-review on the role of physical exercise in promoting anti-inflammatory and neuroprotective effects in MS. Overall, they concluded that exercise intervention studies conducted in mice models of MS are promising, in particular aerobic and strength training regimes in terms of delaying disease onset and reducing the disease severity. They note that while in animal models of MS, most exercise interventions begin before disease initiation and before any clinical sign of disease, studies in humans recruit participants on average nearly a decade after diagnosis and often once disability is established. Thus, intervention studies in early disease cohorts are necessary to estimate the true effect of exercise on the disease course in MS.

The current Research Topic included various approaches and different types of articles which broadened our understanding of this important process in MS. We firmly believe that only through channeling these efforts and their findings we are able to better understand and give a global and adequate overview of the different methods, approaches and fields which are active in the study of neuroplasticity in MS.

